# Teamwork on Patrol: Investigating Teamwork Processes and Underlaying Coordinating Mechanisms in a Police Training Program

**DOI:** 10.3389/fpsyg.2021.702347

**Published:** 2021-09-01

**Authors:** Roar Espevik, Bjørn Helge Johnsen, Evelyn Rose Saus, Sverre Sanden, Olav Kjellevold Olsen

**Affiliations:** ^1^Royal Norwegian Naval Academy, Norwegian Defence University College, Oslo, Norway; ^2^BI Norwegian Business School, University of Bergen, Bergen, Norway

**Keywords:** shared mental models, trust, closed loop communication, training and development, teamwork processes

## Abstract

The Big Five theory suggests that five components in teamwork are essential for team effectiveness in stressful environments. Furthermore, three coordinating mechanisms are claimed to be decisive to upholding and informing vital teamwork processes. Although much research has been conducted into the Big Five theory and its components, to the best of our knowledge, no study has yet been made of the relative importance of the three mechanisms and their impact on team effectiveness. Also, only a few studies have tried to investigate whether the components and the coordinating mechanisms are trainable. This study aims to make a theoretical contribution to the part of the theory focusing on the coordinating mechanisms. Secondly, it investigates whether training can improve team performance. Working in teams of two, 166 police officers participated in a simulated operational scenario. Correlational analyses indicated that all Big Five teamwork behaviors and coordinating mechanisms relate to external ratings of team performance. Only the mechanisms of Closed Loop Communication (CLC) and Shared Mental Model (SMM) predicted performance indicators, with SMM predicting above and beyond the effect of CLC. No effect of the training program was found. The study provides new evidence in a police situation that the most important coordinating mechanism of the Big Five theory is that of shared mental models, which in turn has consequences for the type of training needed.

## Introduction

In the aftermath of July 22, 2011, where a single terrorist killed 77 persons, the Norwegian National Police Directorate concluded that the police force capacity to perform sharp missions had unpredictable situations demand more than basic skills and procedures to be strengthened. Thus, the main object confronting a possible evolving life-threatening situation (e.g., a terrorist) was that the first patrol on site should be better at resolving emergency incidents, when there was no time to wait for force build-up (Politidirektoratet, 2013). Put together this require a focus on what and how to train. Training of police officers has traditionally been executed in a uniform manner, where curriculum and standard scenarios is the chosen form. However, unpredictable situations demand more than basic skills and procedures.

Improvement in this context entails training and Aguilar-Moya et al. (2013) categorized research on police training over a 23-year period (1988–2011) and showed that the most reflected descriptors in published articles were skills and management. “Skills” were often associated with and “mental health.” Accordingly, despite an increase in articles published there seems to be a lack of scientific involvement in police training aimed at resolving violent and unpredictable situations. One important aspect in frontline policing is decision making under conditions of uncertainty and unfortunately this seems to rarely be the focus of training. Thus, there is a growing need for new police training research (Aguilar-Moya et al., 2014).

**Figure 1:**
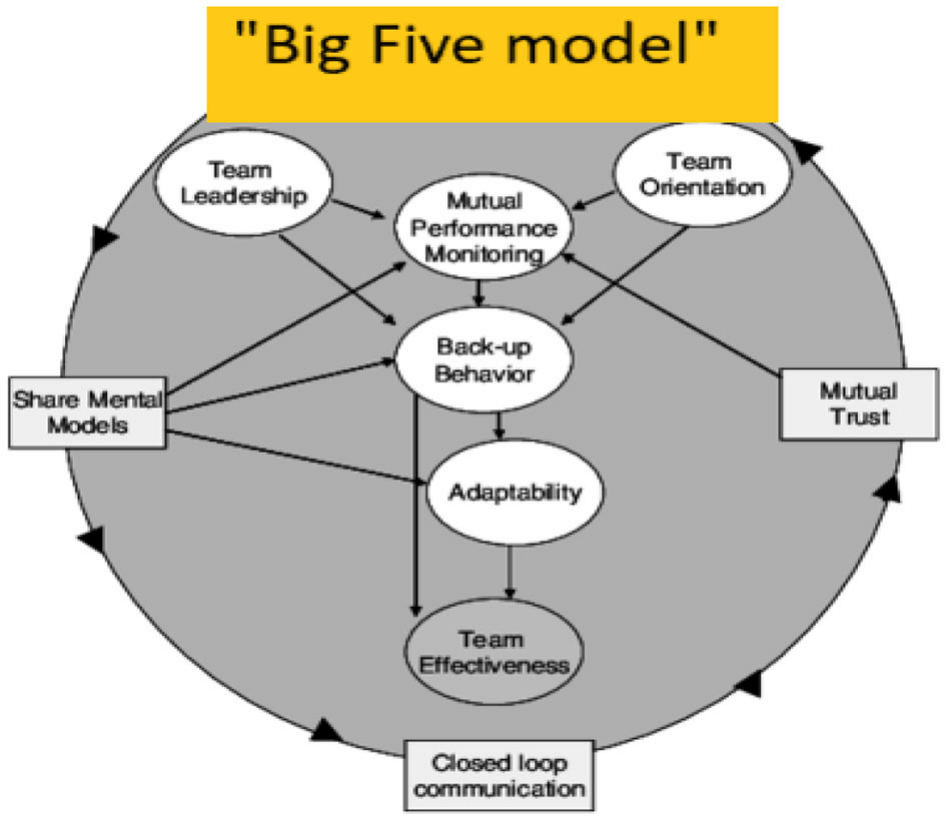
Graphical representation of high-level relationships between the big five and the coordinating mechanisms (Salas et al., 2005).

In Norway, a police patrol normally consists of two police officers. They may be defined as a team since they work toward similar goals and depend on each other to succeed (Stagl et al., 2007). It has been argued that, even if the individual team members are skilled and able, they do not always function as an effective entity (Hackman, 1990; Salas et al., 2005; Hopkin and Wise, 2018). Therefore, the ability of patrols to perform teamwork becomes essential to success, and the study of which and how factors influence the performance of emergency teams is imperative. Examples drawn from the police sector reveal a variety of factors, such as the impact of unit size (Terpstra, 2018), interservice cooperation (Sestoft et al., 2014), training platform for cross-national police (Jaspaert et al., 2019), and team attributes (Schaveling et al., 2017). Proposed mechanisms whereby police teams' performance is enhanced range from a focus on emotional aspects, such as cohesion and familiarity of team members, to cognitive mechanisms, such as the effective utilization of individual team members' knowledge structures (Cotard and Michinov, 2018). Transactive memory components represent an example of the latter (Cotard and Michinov, 2018), in which focus is placed on coordination and specialization within the team and its different team members. To face the situation at hand Salas et al. (2005) based on the notion of shared mental models (Cannon-Bowers et al., 1993) put forward a theory of shared cognitive structures to explain why and how some teams outperform others (see Figure 1).

Despite considerable research interest in teamwork, researchers continue to disagree as to which components subsume teamwork as a construct (Duel, 2010), and how it relates to team effectiveness. Salas et al. (2005) examined 138 teamwork models, and, based on similarities and what could be empirically tested, they proposed five core teamwork components. *Team leadership* entails the ability to direct and coordinate the activities of other team members. *Team orientation* is an attitude characterized by a tendency to take other team members' behavior and input into account during group interaction, and the belief in the importance of team goals over individual team members' goals. *Mutual performance monitoring* involves the ability to apply appropriate task strategies in order to develop a common understanding of the team environment. This again enables *backup behavior*, which entails team members' ability to anticipate each other's needs, through knowledge about their responsibilities, so that they can provide support with the proper action or information. Finally, there is *adaptability*, which concerns the team's ability to adjust team strategies and alter the course of action based on information gathered from the environment through the use of backup behavior and mutual performance monitoring (Salas et al., 2005).

The five teamwork behaviors are claimed to be essential to the promotion of team performance. The Big Five model has received considerable attention by practitioners, especially within the health industry, where several tools to diagnose team deficits or training needs have been developed based on the Big Five theory (e.g., TeamSTEPPS; Cooke, 2016; Weld et al., 2016). We have found only two empirical studies (Johnsen et al., 2016, 2019) investigating the Big Five within the police domain. Both indicated that police officers accede to the Big Five theory (i.e., high on perceived learning and relevance).

However, to work effectively as one team, team members must know their roles in the task, of the resources available, each other's capabilities and able to communicate freely and clearly. Hence, Salas et al. (2005) proposed three coordinating mechanisms as necessary prerequisites to ensure that the five teamwork behaviors are consistently updated, and that relevant information is distributed throughout the team. First, shared mental models (SMM), which are defined as an organized knowledge structure of the relationship among the tasks the team is engaged in and how the team members will interact (Cannon-Bowers et al., 1993). Secondly, closed loop communication (CLC), defined as the exchanging of information and coordinating actions through explicitly expressing feedback and response (McIntyre and Salas, 1995). Finally, mutual trust is when team members perceive intentions behind feedback as positive, so that all team members freely share information without process loss (Steiner, 1972). All three coordinating mechanisms are claimed to be decisive tools for upholding and informing the Big Five teamwork processes. However, the three underlying and theoretically based coordinating mechanisms have, to the best of our knowledge, not been collectively, empirically tested. Although there is much research that has been conducted into the Big Five theory and its components, to the best of our knowledge, no study has yet been conducted that investigates the relative importance of the three mechanisms on their impact on team effectiveness. Research supports the existence of these constructs when they are investigated separately, in pairs, or together with one or two of the five teamwork components (e.g., backup behavior and SMM, Schmidt et al., 2014). Notably, the three coordinating mechanisms may be intercorrelated and dependent on each other or vary in their importance. The relative importance of these mechanisms has theoretical and practical consequences and may implicate a different type or focus when training to improve teamwork skills. Few studies have tried to investigate whether the five components of teamwork and the three coordinating mechanisms are trainable.

**Figure 2:**
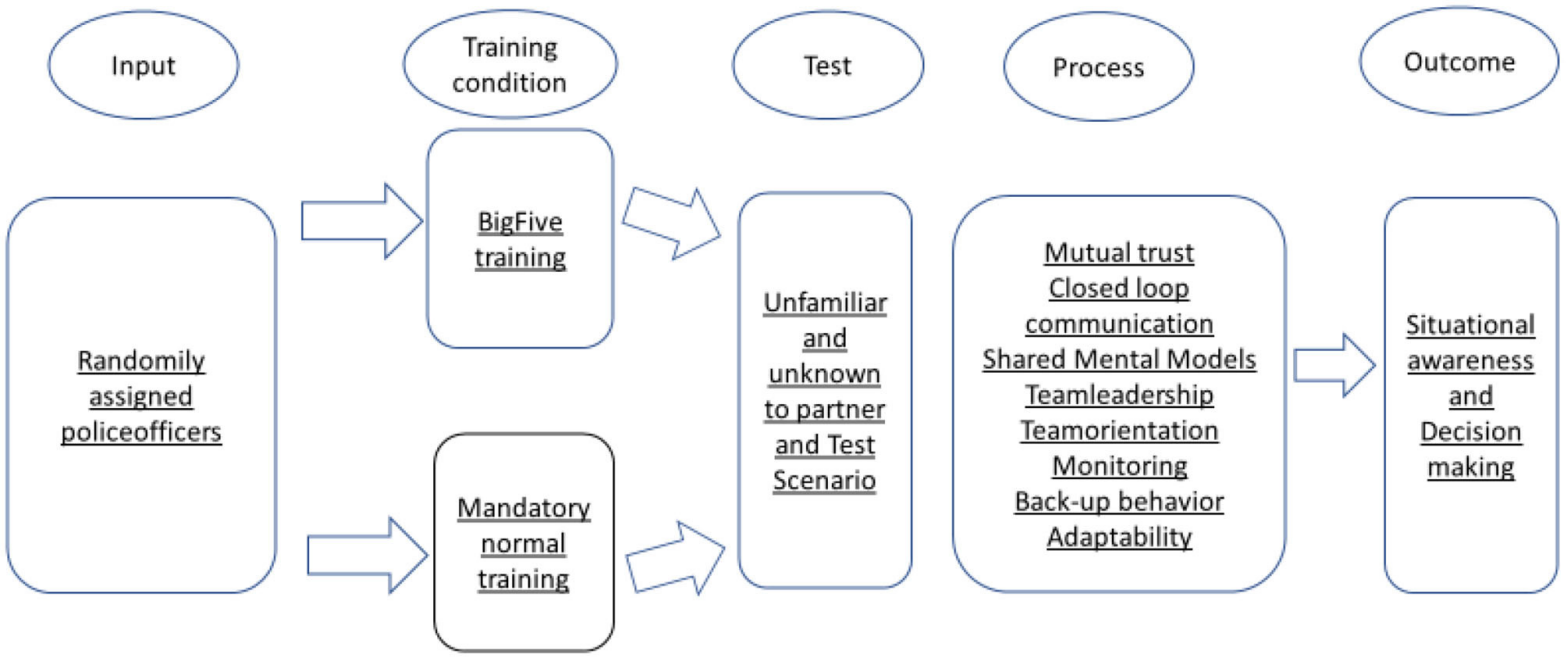
Graphical representation of the research process using a Input-Process-Outcome design.

Accordingly, the study has two aims. First, it intends to give a theoretical contribution to the Big Five theory by investigating the relative contribution of the coordinating mechanisms in predicting team performance. Secondly, it investigates whether a brief intensive training program can improve both the mechanisms and the five teamwork behaviors (see Figure 2).

### Theoretical Background

#### Coordinating Mechanisms

Cannon-Bowers and Salas (1998) argue that, to enable a team to adapt effectively in a dynamic, stressful situation, team members must be able to predict what others in the team will do and what they are going to need in order to execute their intended behavior. The coordinating mechanism Shared mental models (SMM) is proposed to meet such a need. SMM is drawn from theories of individual mental models used to explicate individual cognitive functioning or understanding. At the individual level, mental models refer to a structure of known elements (e.g., declarative knowledge) and the relationship between those elements (Shavelson, 1974). These structures serve as mechanisms that people use in order to describe the purpose and form of a system, as well as its functioning in its present and future state (Rouse and Morris, 1986). Cannon-Bowers and Salas (1990) proposed extending the concept of individual mental models to the team performance domain, hypothesizing that team performance is a function of the extent to which members held similarly organized expectations in relation to the task or each other. SMM are defined as a shared organized understanding and mental representation of key elements of the team's relevant environment. These SMM enable team members to form accurate explanations and expectations of the task. This will in turn enable team members to coordinate their actions and adapt their behavior to the demands of the task and to other team members (Cannon-Bowers et al., 1993). SMM are assumed to enable team members to predict task needs and the actions of other team members, and thus enable them to adapt their own behavior accordingly without communicating explicitly.

A number of studies have indicated that SMM contributes to increased team effectiveness, such as better communication strategies and, in general, an increased effectiveness (e.g., Urban et al., 1995; Volpe et al., 1996). Stout et al. (1999) reported that better SMM resulted in better communication strategies and fewer errors. Furthermore, in a study of simulated anti-air warfare, Mathieu et al. (2000) reported that SMM was related to better accuracy, increased survival, and higher numbers of enemy aircraft shot down. “One challenge for the SMM concept is that at least seven terms have been used to define shared cognitive structures (e.g., shared cognition, teammind, teamthink, team cognition, and shared member schemas; Rentsch and Davenport, 2006). Accordingly do Ward and Eccles (2006) claim SMM too be too theoretical and that more empirical documentation on its foundations is needed.”

In order to coordinate effectively, correct information must be distributed within the team (Salas et al., 2005). Therefore, based on McIntyre and Salas (1995), the second coordinating mechanism Closed loop communication CLC was proposed. CLC is a communication model riginating from military radio transmissions based on verbal feedback to ensure proper team understanding of a meaningful message. CLC is a three-step process, where (1) the transmitter communicates a message to the intended receiver, utilizing their name when possible, (2) the receiver accepts the message with acknowledgment of receipt via verbal confirmation, seeking clarification if required, and (3) the original transmitter verifies that the message has been received and correctly interpreted, thereby closing the loop (Burke et al., 2004).

In a recent study of patient safety, better CLC was significantly and negatively associated with the number of critical incidents (Lacson et al., 2016). El-Shafy et al. (2018) further underlined the importance in a study of trauma team leaders that showed that CLC prevented medical errors, and also how it increased working speed and efficiency in pediatric trauma resuscitation. But there are also concerns as Salik and Ashurst (2020) points out, if all team members constantly initiate CLC, communication overload can result in a lack of leadership and delayed patient assessment and intervention. Another limitation is that different professions have different expectations regarding the content, timing, and generalized structure of information transfer, and may not grasp the roles and priorities of others (Smith et al., 2008). Another study of 16 emergency trauma teams showed that, despite a focus on the importance of CLC, the difficulty in achieving safe and reliable verbal communication within the interdisciplinary team remained (Härgestam et al., 2013). The study concluded that validated training models were called for, combined with further implementation studies. Espevik et al. (2011) showed that naval teams with superior SMM in a simulated operation also exhibit more CLC and perform better when faced with a novel and unknown situation.

A team also has to concentrate as much as possible on the task at hand without using cognitive and physical resources on tasks based on wrong or misunderstood intentions (Bandow, 2001; Webber, 2002). Therefore, the last proposed coordination mechanism is *mutual trust*. Ayenew et al. (2015) linked trust to superior safety performance in nuclear power plants. However, a meta-analysis also revealed a dual side of trust, where high levels of trust could lead to team members becoming too comfortable and less safe, whereas low trust might lead them to avoid the collaboration necessary to be safe (Breuer et al., 2016). Based on this, there seems to be a need for more research into training and intercorrelation between the coordinating mechanisms.

Salas et al. (2005) posits that the three coordinating mechanisms predict to which degree five teamwork components are up-to-date and correct information is distributed throughout the team. As such, all three must be understood as decisive prerequisites for the effect of the five teamwork behaviors. Further, they argue that the teamwork behaviors relate directly to performance. This is claimed to be because the increased sharing of information, the even distribution of workload, and the high level of coordination and monitoring increase the team's perception of the dynamic aspects of their surroundings. This again enables them to develop, consider, and evaluate different courses of action and finally act on the best of them. However, one challenge seems to remain unaddressed: the relative importance of the three mechanisms in their impact on team effectiveness has not been empirically investigated. This could have significant implications for the design of training and exercises.

#### Teamwork Behaviors

Since the Big Five model was presented in 2005, several studies have added to the original embedded empirical evidence that the Big Five behaviors were connected to performance (e.g., backup, Fincannon et al., 2008; monitoring, Albon and Jewels, 2014; adaptability, Uitdewilligen et al., 2018). Other studies showed similar connections, but few of them took the entire Big Five model into consideration when some of the components were studied. Therefore, most of the studies have investigated the five team processes by combining different team processes as well as organizational factors (e.g., staffing or structure). To the best of our knowledge, there are few studies that have investigated the effect of all five processes and the underlying coordinating mechanisms within the same study. One exception to this is Kalisch et al. (2009), who studied both the team processes and the coordinating mechanisms on nursing teams. These researchers utilized a qualitative method, leaving an open question of empirical quantitative support for the proposed team processes.

#### Study Aims

Within the police community, there were surprisingly few empirical reports using the five teamwork approach. One exception reported high levels of face validity when police officers rated a training program focusing on the Big Five theory. They also reported high levels of perceived relevance and learning effects of the training (Johnsen et al., 2016). Another case study discussed SMM and the five behavioral processes within a police context (Johnsen et al., 2019).

However, the paper focused on the introduction of the five teamwork behaviors as a tool in the selection process of police special forces and in the evaluation of training scenarios. Being a case-study, it has obvious limitations regarding the generalizability of its conclusions. In order to argue for the use of Big Five teamwork as a useable model in the operational police domain, a relationship must be established for each teamwork behavior and team performance (Hayes, 2018). One theoretical contribution is to investigate whether all coordinating mechanisms and Big Five teamwork behaviors are intercorrelated, and whether all the team processes are related to team performance. We therefore anticipate (H1) that all three coordinating mechanisms and five teamwork behaviors based on the Salas et al. (2005) model will relate positively to Situation Awareness (SA) and decision-making in the police domain.

Although it is a crucial element of the proposed model, Salas et al. (2005) do not give any empirically based argument for the relationship between the three coordinating mechanisms and team effectiveness. It is therefore important to investigate to what degree the three coordinating mechanisms explain the performance of teams in the sense of the team's shared SA and decision-making behavior. Accordingly, three coordinating mechanisms were suggested, and different authors have emphasized and studied them differently. As mentioned, a study of teamwork in pediatric trauma resuscitation showed that CLC prevented medical errors (El-Shafy et al., 2018). Furthermore, in a study of software teams, trust and SMM were claimed to be of fundamental importance (Moe et al., 2010), and the two factors that made teams of naval cadets outperform other teams were better SMM and the ability to use CLC when they met an unknown situation (Espevik et al., 2011). None of these provide any evidence of the relationship between the three coordinating mechanisms, and the research only confirms what Salas et al. (2005) proposed—i.e., that they are all important.

Furthermore, Salas et al. (2005) deduce from a review of the literature that both trust and SMM are vital to mutual performance monitoring behavior. CLC is assumed to play a part in all coordinating mechanisms and Big Five teamwork processes. Salas et al. (2005) do not argue for the relative contributions of the mechanisms but mainly focus on the function of these (ensuring that information is distributed in the team). According to the initial proposal, trust only plays a part in how mutual monitoring behavior is understood and operates more as a barrier that hinders good teamwork if absent. SMM is a cognitive concept and enables the team to monitor, support and adapt more correctly. Theoretically, SMM is thought of as the core coordinating mechanism, and is viewed as being both the aim and basis for the Big Five teamwork processes. However, there are no empirical studies to back up such a claim. Therefore, we anticipate (H2a) a positive effect of each of the coordinating mechanisms on SA and decision-making, and (H2b) that an effect of SMM would be present even after controlling for both trust and CLC.

The importance of learning and continuous improvement will increase when a police patrol faces uncertain and unclear situations. The Big Five teamwork behaviors, with the three coordinating mechanisms, claim to respond to this. Accordingly, this paper finally aims to investigate whether the Big Five processes and coordinating mechanisms are trainable using a brief training program. A training program for frontline police officers that is perceived as relevant and with established learning effects, focusing on strengthening teamwork behaviors, tries to respond to this aim (see Johnsen et al., 2016 for an outline). We hypothesize (H3) that a training effect would occur with the trained group showing higher ratings of coordinating mechanisms, teamwork behaviors, and finally on SA and decision-making, compared to an untrained group.

## Methods

### Subjects

A total of 166 police response personnel (30 females and 136 males) performing in teams of two (83 pairs) participated in the study. All subjects were employed by the West police district in Norway, which has a total of 1,300 employees, including civilian and non-operational personnel. A subsample of 27 of these teams (10 females and 44 males) also received the brief training program. The present study utilized the same sample as presented in an earlier study (Johnsen et al., 2017), and consisted of both urban and rural police officers as well as a variety of main functions. The functions included patrol officers, police dog handling, investigation, and officers attached to task forces for organized crime. The age of the subjects was categorized as below 25 years (4.9%), between 25 and 29 years (23.5%), between 30 and 39 years (41.5%), and between 40 and 57 years (30.1%). The sample consisted of 4.9% officers with <1 year of active duty, 22.8% who reported 2–5 years of experience, 42.6% who had been in the force for between 6 and 10 years, and 29.6% who reported having been a police officer for between 11 and 20 years.

### Questionnaires

Based on the Salas et al. (2005) definition of the “Big Five” teamwork behaviors and the three coordinating mechanisms, a questionnaire for observer ratings by Subject Matter Experts (SME) was developed. Two SMEs initially rated all “Big Five” teamwork behaviors and coordinating mechanisms independently, and after each test they made a consensus-based decision for the patrol.

“*Big Five” teamwork behaviors* were rated from unacceptable (1) to exceptional (7) by the following statements from Salas et al. (2005):

*Team leadership*, the patrol effectively solved team problems (team roles and responsibilities were distributed in the team).
*Team orientation*, the goals of the patrol were placed above those of the individual (showed a high degree of involvement, participated actively, and showed good attitudes).
*Mutual performance monitoring*, the patrol adjusted and reinforced each other (feedback when wrong or right was accepted and implemented by team members).
*Backup behavior*, the patrol showed a high degree of backup behavior (team members helped/assisted without being asked, pushing of information).
*Adaptability*, the team showed the ability to adjust strategies (they had dynamic coordination to meet shifting internal and external needs).
*Coordinating mechanisms* were rated from unacceptable (1) to exceptional (7) by the following statements from Salas et al. (2005):
*Mutual trust*, the members of the patrol trusted each other (understanding and acceptance that feedback was intended to improve performance).
*Closed loop communication (CLC)*, the patrol exchanged information, and coordinated actions through feedback and response.
*Shared mental model (SMM)*, the patrol showed an ability to create a common understanding for the mission, and updated each other on the priorities and situation.
*Team performance indicators*. This variable was used as an outcome measure and entailed situation awareness and decision-making behavior. The patrol's ability to create SA (Endsley, 1995) was measured by an aggregated score of two questions: *the patrol discovered changes and mismatches in the situation* (i.e., level 1) and *the patrol showed an ability to keep an updated and correct picture of the situation at hand* (level 2). Criteria-based evaluation of decision-making behavior consisted of accuracy, latency, and mission effectiveness (Cannon-Bowers and Salas, 1998) and was measured as the ability to evaluate, act, and the degree of solving the mission. Decision-making behavior was measured by an aggregated score of three questions: *the patrol showed an ability (a) to evaluate different courses of action (based on communication between team members, reports to the dispatch, adaptation of distances, preparation to use different aids, etc.), (b) to act (performance of tactics), and (c) to successfully accomplish its task/mission (handling of persons, control of the situation, adequate use of force, prevention of possible injuries etc.)*. Since the focus of the present study was on team performance, no individual scores were used in the analyses.

#### Procedure

Before the start of the experiment, the participants read and signed an informed consent statement. They received information about their rights to leave the study at any time. No participants withdrew from the study, although some participants from the training group did not conduct the test scenario due to other police duties.

The participants were assigned to two groups. One group underwent the outlined training program at the Royal Norwegian Naval Academy. Personnel were randomly assigned to the trained or control condition. The allocation to groups was performed by the leaders of the training wing. Neither the observers nor the participants were informed of whether the enrollment was in a trained or a non-trained group. Two experienced police officers attached to the training wing, who are also engaged in regional police training on a daily basis, were used as subject matter experts (SMEs). Each of the SMEs had more than 20 years of service in the police force. Instructors attached to the training wing occupied this role based on their knowledge of police tactics and their ability to observe and guide colleagues (see Lavin et al., 2007, for a critical discussion of the use of SMEs). The leaders of the training wing were also involved in designing the study, including the variables used. The same SMEs observed and rated all 83 patrols. The SMEs were located in the same room about 10 feet from the participants with no obstruction to their view of the scenario. However, one exception from this was the driving phase for the patrol, where, due to practical reasons, the observers were unable to observe the patrol. The SMEs were blinded to which teams were in the two training conditions. Only the consensus scores were recorded. The SMEs' evaluations were filled in immediately before the debriefing of the police officers. Since the test was performed as part of the annual training, the presence of the SMEs following the execution of the scenario was consistent with standard procedure during training. This, together with the time passed since the training program was carried out, minimizes the possibility of priming the trained group.

*Test situation*. The test was executed at the police training facility as a part of their annual retraining program. The criteria for developing the scenario were that it should be realistic, operationally relevant, critical (by posing a threat to the officers, perpetrator and civilians), as well as “foggy,” in order to induce variation in SA and decision-making. All police officers were randomly assigned to a patrol. None of the teams consisted of members that regularly conducted operational patrols together.

The instructions were given orally while the subjects were seated in a patrol car, and consisted of a verbal report from the dispatch central. The message was that a robbery had taken place and that a knife had been used. This showed willingness to use deadly force. The perpetrator was observed entering a hostel known for harboring several previously convicted persons. The mission was to guard the back door while another unit entered through the front door. The drive to the hostel took 5 min. During this period, the subjects were seated in the car, preparing themselves for the task at hand. No restrictions on the officers were communicated, and an order for armament was issued. Since the Norwegian police is unarmed, the standard procedure involves the storing of weapons (handgun and MP5) in the patrol car, in addition to heavy body-armor and an armored shield. Thus, the officers were armed with a sidearm, baton, pepper-spray, and light body-armor. Heavy body-armor (including helmet with visor), a shield and an MP5 were optional. After positioning themselves at the back door, two persons would exit through the door. The first person was similar to the description of the perpetrator except for two minor, but critical, features. The color of his pants was light gray instead of black, and he carried a short umbrella (not a knife). This manipulation was designed in order to have a salient inject tapping into levels one and two of Endsley's (1995) model of SA. The second person, the perpetrator, would come through the door 30 s later. He had one hand in his pocket, and during the interaction he would take it out holding the knife, and further threaten the officers. The task of the patrol was to handle both situations, almost (30 s) simultaneously.

*The training program*. The trained group received an 8-h training program in advance of the test scenario. The program focused on SA, Big Five team processes, and the coordinating mechanisms, mutual trust and communication in teams and decision-making. The time was divided, allocating 3 h both for SA and team training, respectively. The remaining 2 h were allocated to personal reflection and a scenario at the shooting range executed individually. An extensive outline of the theoretical foundations and the training program is described in Johnsen et al. (2016).

SA and Big Five teamwork training consisted of video simulation that included a freeze technique (Flin et al., 2008). Significant focus was placed on the detection of critical elements (SA Level 1). The participants were also challenged to use Big Five teamwork categories and suggest what they, at this stage, wanted to inform or agree upon with their partner. Lectures in SA, “Big Five” teamwork, and coordinating mechanism behavior were held.

In order to enhance team performance, a lecture based on the Salas et al. (2005) model was delivered, followed by a practically oriented group session. An extensive outline of the theoretical foundations and the training program is described in Johnsen et al. (2016). The group was placed in a situation with time constraints, where they were blindfolded and challenged to solve an unfamiliar problem. One of the team members acted as team leader, and was unable to view the process of executing the task, only communicating to the rest of the team through an intercom. One of the main focuses of the training was to exemplify the pushing of information. By separating the leader from the team, the team is forced to push information to the leader, and blindfolding the team members presents an opportunity to highlight the importance of the pushing of information to the team members. After a predetermined period of time, one of the blindfolded team members was secretly given vital information, which, if the other team members responded according to the Big Five theory, would enable the team to solve the problem. The importance of planning (i.e., assigning roles and responsibilities), information exchange (pulling and pushing of information), and monitoring was emphasized during the training session. This was viewed as crucial in scenarios with both a short and long time-frame.

#### Design and Statistics

Group differences between the subsample exposed to training and controls were tested using *t*-test for independent samples. The relationship between team behavior and performance was tested by means of Pearson product moment correlation (H1). The relative contributions of coordinating mechanisms on team effectiveness were tested using multiple regression (enter methods; H2a/b). Multiple regression is a suitable method for studying separate and collective contributions of one or more independent variables on the variation of a dependent variable (Wampold and Freund, 1987). Results from the multiple regression were followed up using hierarchal regression (H2b). Only variables contributing significantly in the multiple regression were included in the hierarchal analysis. The results of the regressions analysis are presented as both unstandardized and standardized effects and adjusted R was used in order to present magnitude of explained variance.

The relationship between team behavior and performance was investigated by means of Pearson product moment correlation. The contributions of coordinating mechanisms and team behavior on team performance indicators were calculated using multiple regression (predictor variables were entered in one block). The Variance Inflation Factor (VIF) was calculated as an index of multicollinearity. This index is one of the most common tools used to determine the inflation in the variances of the parameter estimates due to multicollinearity caused by correlated independent variables (Vatcheva et al., 2016). Although no exact cut-off point has been established, a common practice is to consider VIFs <10 as acceptable (Kutner et al., 2004; Vatcheva et al., 2016). The collinearity statistics showed all VIFs being within this criterion for both the dependent measure of SA and decision-making behavior. The collinearity diagnostics using SA as a dependent variable showed adaptability with the lowest (VIF = 5.84) and support behavior with the highest index (VIF = 8.52). When decision-making behavior was used as an outcome measure, SMM revealed the lowest (VIF = 5.29) and team orientation showed the highest coefficient (VIF = 9.56). Results from the multiple regression were followed up by means of hierarchical regression with the aim of calculating the relative contributions that the separate coordinating mechanisms exerted on performance indicators. Only variables contributing significantly in the multiple regression were included in the hierarchical analysis. The results of the regressions analysis are presented as both unstandardized (*B*) and standardized effects (in tables), and adjusted R was used in order to present magnitude of explained variance. Group differences were explored using *t*-test for independent samples. All statistics were performed using SPSS version 25.

## Results

### Correlational Analyses

In order to explore if all three coordinating mechanisms and five teamwork behaviors relate positively to performance indicators (H1), the correlational analyses revealed significant intercorrelations for all measures included in the analyses (see Table 1 for a detailed description of coefficients and significance levels). Regarding performance indicators, both the lowest and strongest coefficients were found for Decision Making Behavior (see Table 1 for a detailed description of coefficients and significance levels).

**Table 1:** Means (M), Standard Deviations (SD), and Intercorrelations between the coordinating mechanisms, the big-five teamwork processes and performance indicators.

**Team process**	**1**	**2**	**3**	**4**	**5**	**6**	**7**	**8**	**M**	**SD**
1. Team leadership	–								4.19	0.92
2.Team orientation	0.89^**^	–							4.37	0.89
3.Monitoring	0.77^**^	0.83^**^	–						4.14	0.98
4.Backup behavior	0.78^**^	0.86^**^	0.92^**^	–					4.13	1.06
5.Adaptability	0.79^**^	0.83^**^	0.83^**^	0.84^**^	–				4.11	1.00
**Coordinating mechanism**										
6.Mutual trust	0.90^**^	0.87^**^	0.78^**^	0.80^**^	0.82^**^	–			4.33	0.80
7.Closed loop communication	0.82^**^	0.85^**^	0.87^**^	0.87^**^	0.86^**^	0.83^**^	–		4.14	1.08
8.Shared mental models	0.87^**^	0.86^**^	0.76^**^	0.76^**^	0.84^**^	0.89^**^	0.79^**^	–	4.23	0.86
**Performance indicator**										
9.Situational awareness	0.84^**^	0.86^**^	0.88^**^	0.89^**^	0.89^**^	0.83^**^	0.88^**^	0.83^**^	4.12	0.96
10.Decision-making behavior	0.80^**^	0.85^**^	0.90^**^	0.89^**^	0.87^**^	0.80^**^	0.86^**^	0.81^**^	23.24	3.68

** *p < 0.01*.

### Regressing Situation Awareness onto “Big Five” Teamwork Behaviors

When regressing SA on the “Big Five” teamwork behaviors (H2a), the results of the analysis revealed a significant model (*F* = 116.53, *p* < 0.001). The model explained 87.6% of the variance in SA. However, the regression analysis showed that only team leadership, backup behavior, and adaptability were significantly related to SA. The same analysis revealed no significant relationship when SA was regressed on team orientation and monitoring (see Table 2 for unstandardized and standardized effects, as well as *t*-values and significance levels).

**Table 2:** Unstandardized and standardized coefficient (β), standard error, *t*-values, and significance levels for situation awareness regressed upon team behavior.

**Team behavior**	**Unstardadized beta**	**Standard error**	**β**	***t*** **-value**	**Significance level**
Team orientation	0.01	0.22	0.01	0.04	0.967
Team leadership	0.42	0.18	0.21	2.39	0.019
Monitoring	0.35	0.19	0.19	1.86	0.067
Backup behavior	0.48	0.19	0.28	2.49	0.015
Adaptability	0.59	0.15	0.33	4.02	0.000

### Regressing Decision-Making Behavior Onto Big Five Teamwork Behaviors

When regressing decision-making behavior on the Big Five teamwork behaviors, a significant model occurred (*F* = 105.52, *p* < *0.001*). The model explained 87.3% of the variance in decision-making behavior. As can be seen in Table 3, the regression analysis revealed significant effects only for monitoring and adaptability. No relationship was found when SA was regressed on team orientation, team leadership, and backup behavior (see Table 3 for details).

**Table 3:** Unstandardized and standardized coefficient (β), standard error, *t*-values, and significance levels for decision-making behavior regressed upon team behavior.

**Team behavior**	**Unstardadized beta**	**Standard error**	**β**	***t*** **-value**	**Significance level**
Team orientation	0.21	0.38	0.06	0.56	0.578
Team leadership	0.27	0.30	0.08	0.88	0.382
Monitoring	1.22	0.32	0.40	3.77	0.000
Backup behavior	0.55	0.33	0.20	1.68	0.097
Adaptability	0.75	0.25	0.25	2.98	0.004

### Regressing Situation Awareness Onto Coordinating Mechanisms

A significant model including all three mechanisms (H2b) occurred when regressing SA onto the coordinating mechanisms (*F* = 123.73, *p* < 0.001). The model explained 81.8% of the variance in SA scores. Significant effects were found only for CLC and SMM. Therefore, no relationship was found when the SA were regressed on mutual trust (see Table 4 for details of effects, error terms, and significance levels).

**Table 4:** Unstandardized and standardized coefficient (β), standard error, *t*-values, and significance levels for situation awareness regressed upon coordinating mechanisms.

**Coordinating mechanism**	**Unstardadized beta**	**Standard error**	**β**	***t*** **-value**	**Significance level**
Mutual trust	0.21	0.26	0.09	0.81	0.421
Closed loop communication	0.96	0.15	0.57	6.54	0.000
Shared Mental Models	0.63	0.22	0.30	2.88	0.005

### No Further Regression Analysis Including Trust Was Therefore Performed

In order to investigate the relative contribution for each of the coordinating mechanisms in explaining the variance in SA scores, a hierarchical regression analysis was performed. The results showed a significant effect of both CLC and SMM. In the first step, CLC was entered as an independent variable. A significant model occurred [*F*_(83)_ = 280.26, *p* < 0.001], explaining 77.6% of the variance. In the second step, SMM was entered in addition to CLC.

In step two, the significant model [*F*_(83)_ = 186.06, *p* < 0.001] explained an additional 4.7% of the variance. Also in this model, CLC revealed a significant contribution in explaining the variance in SA scores. When adding the independent variable of SMM, a significant relationship was found indicating a unique effect of SMM on SA, even when controlling for CLC (see Table 5 for details).

**Table 5:** Hierarchical regression analyses of situation awareness regressed upon closed loop communication and shared mental model.

**Model**	**Team behavior**	**Unstardadized beta**	**Standard error**	**β**	***t*** **-value**	**Significance level**
1	Closed loop communication	1.48	0.09	0.88	16.74	0.000
2	Closed loop Communication	1.01	0.28	0.60	7.87	0.000
	Shared Mental Models	0.75	0.16	0.35	4.62	0.000

### Regressing Decision-Making Behavior Onto Coordinating Mechanisms

When regressing decision-making behavior on coordinating mechanisms of the theory, a significant model including all three mechanisms was found (*F* = 94.16, *p* < 0.001). The model explained 77.3% of the variance in decision-making behavior. Also in this analysis, the only significant predictors were CLC and SMM. As for the analysis of SA, no relationship was found when the performance indicator of decision-making behavior was regressed on trust (see Table 6 for details of effects, error terms, and significant levels).

**Table 6:** Unstandardized and standardized coefficient (β), standard error, *t*-values, and significance levels for decision-making behavior regressed upon coordinating mechanisms.

**Team behavior**	**Unstardadized beta**	**Standard error**	**β**	***t*** **-value**	**Significance level**
Closed loop Communication	1.53	0.27	0.56	5.74	0.000
Mutual trust	0.22	0.48	0.06	0.46	0.645
Shared Mental Model	1.10	0.40	0.32	2.74	0.008

### No Further Regression Analysis Including Trust Was Therefore Performed

In order to follow up on the regression results and to further investigate the relative contribution of the separate coordinating mechanisms, a hierarchical analysis identical to that for SA was performed. In the first step, CLC was entered, resulting in a significant model [*F*_(83)_ = 223.44, *p* < 0.001), explaining 73.1% of the variance in decision-making behavior. In the second step, SMM was added to the model.

Step two also revealed a significant model [*F*_(83)_ = 17.13, *p* < 0.001], which explained an additional 4.7% of the variance. CLC also revealed a significant effect in this model. In addition, step two exposed the independent variable of SMM as a significant contributor. Therefore, when using decision-making behavior as performance indicator, and controlling for CLC, a unique effect of SMM was found (see Table 7 for details).

**Table 7:** Hierarchical regression analyses of decision-making behavior regressed on closed loop communication and shared mental model.

**Model**	**Team behavior**	**Unstardadized beta**	**Standard error**	**β**	***t*** **-value**	**Significance level**
1	Closed loop Communication	2.36	0.16	0.86	14.95	0.000
2	Closed loop Communication	1.59	0.23	0.58	6.80	0.000
	Shared Mental Model	1.22	0.30	0.35	4.14	0.000

Ten *t*-tests were conducted in order to test the effects of the brief training program (H3), using team performance, as well as all Big Five team processes, and all three coordinating mechanisms as dependent variables. The results showed no significant difference between the untrained and trained groups for either of the variables studied.

## Discussion

According to this study, all Big Five teamwork behaviors and the coordinating mechanisms seem to correlate with external ratings of team performance indicators (i.e., SA and DM). The regression results showed that four of the five teamwork behaviors were related to either SA or decision-making behavior. Surprisingly, team orientation did not show any significant effect. The results from the regression analyses showed that the coordinating mechanisms of CLC and SMM predicted team performance, with SMM predicting above and beyond the effect of CLC. Contrary to this, no significant effect occurred when measures of performance were regressed upon trust. No effect of the training program occurred since the trained group did not show more of the Big Five teamwork behavioral markers or coordinating mechanisms, nor better performance compared to the untrained group.

### Big Five Teamwork Theory and Performance Indicators

Our first hypothesis was to explore whether all components were positively relevant to team performance indicators. The use of dependent measures of SA (Endsley, 1995) and decision-making behavior (Cannon-Bowers and Salas, 1998) revealed a correlation between all elements in the model and the indicators of performance used. In addition, four of the five teamwork behaviors explained a variance in SA, decision-making or both. Only team orientation failed to do so. This is the first study using a quantitative approach to show that all Big Five teamwork behaviors and the coordinating mechanisms seem to be connected to performance (see Kalisch et al., 2009 for an exception). Furthermore, the coordinating mechanisms were highly intercorrelated. On a theoretical level, this lends support to the proposed model. On a practical level, it could be argued that a police patrol that executes all Big Five teamwork behaviors seems to be more able to perceive and comprehend the situation the team is facing. However, caution should be exercised since this conclusion is based on correlational analysis. Also, the results of analyses of the three coordinating mechanisms are in line with the Big Five teamwork behaviors. It was interpreted that they provide essential coordination to secure team output by being highly correlated with the Big Five team processes. Therefore, we argue that this provides new evidence that all the teamwork behaviors within the Big Five theory ensure that a team, in a stressful new, ambiguous and unclear operational situation, is more efficient. This goes beyond previous findings, where the focus seems to be on one or two teamwork behaviors and not the entire Big Five theory. This study is therefore the first to give empirical, quantitative evidence that the performance of police patrols is related to whether and how they carry out Big Five teamwork behaviors and coordinating mechanisms. This could be generalized for other teams that have to deal with uncertainty in high stress situations (e.g., military personnel, firefighters, or health workers in an ongoing emergency situation).

### Importance of Coordinating Mechanisms

The notion that teamwork is associated with team performance is hardly revolutionary. However, Salas et al. (2005) suggested three coordinating mechanisms, which do not determine how inputs are incorporated, but ensure that the Big Five teamwork components are consistently updated and that relevant information is distributed throughout the team. In our view, this is the most noteworthy and novel part of the Big Five theory approach to teamwork. The predictive power of the coordinating mechanisms on team performance has attracted little attention and, as such, was the main focus of the present paper.

Contrary to our expectation, trust did not explain any variance in either SA or decision- making behavior. The importance of different levels of trust in teams has been noted by McComb et al. (2017). In a quantitative study, trust and SMM were tested for group differences between nurses and physicians. Differences in perceived role responsibilities were interpreted as being low SMM but, more interestingly, nurses trusted physicians more than vice versa. Although McComb et al. (2017) measured SMM and trust, they did not test the model in relation to performance. However, we find it interesting that both professions showed a high level of trust toward other members of their own profession. Therefore, there may be similarly high levels of trust between police officers, as they also understand themselves as belonging to a profession with a certain expertise, responsibility and collectiveness (Huntington, 1981).

Trust could be viewed as a belief system, and it is defined as a willingness to be exposed to vulnerable situations as a consequence of others' decisions or behavior because one expects these to be well-intended (Olsen et al., 2020). This belief system could impact the Big Five team processes by means of increased information sharing, coordination, and a willingness to listen to and support other team members. The correlation between trust and team performance indicators did not hold up in the regression analyses, indicating that the effect was caused by other variables not controlled for in the present study. The same line of thinking may apply for the Big Five teamwork behaviors, with team orientation failing to explain any variance in performance indicators. Salas et al. (2005) argue that team orientation is an attitude, and one explanation could be that team orientation involves some of the same properties as trust. Therefore, team orientation is also part of a belief system, where one expects other police officers to have good intentions. Accordingly, one could argue that police officers take for granted that other police officers value the goals of the patrol over those of the individual (i.e., definition of team orientation). We suggest that trust and team orientation are addressed in future research concerning bases for swift trust, attitudes, beliefs, and behavior within professions.

On the other hand, CLC predicted team performance indicators. This is in line with Espevik et al. (2011), who showed that superior-functioning teams of naval cadets met a new and uncertain situation with CLC. Also in the present study, the test situation placed each patrol in a new and uncertain situation, including unknown team members. Therefore, this study provides evidence that CLC is an important coordinating mechanism to update all Big Five teamwork behaviors, and in a way that results in better SA and decision-making behavior.

The coordinating mechanisms CLC and SMM are both mechanisms related to generating, maintaining, and altering knowledge structures in the team. This is performed by describing, clarifying, and projecting into the future, and, as this study shows, is decisive for a police patrol. Salas et al. (2005) argue that the importance of SMM and CLC increases when teams must perform in stressful conditions. However, in the present study, SMM predicted team performance even when controlling for CLC. This indicates that, even if both mechanisms are important, SMM seems to be more crucial. SMM represents an explanation for how the environment is functioning, whilst CLC represents the ability to get this understanding across to all team members (Espevik et al., 2011). CLC contributes to these knowledge structures by questioning and confirming the reality of the SMM currently existing in the team, causing an interaction between CLC and SMM. However, without a shared understanding (i.e., SMM), CLC has less to contribute because explicitly sending and receiving information becomes pointless without prior understanding. This interaction could be a prerequisite for increased performance by the team. Therefore, CLC and SMM increase performance by generating and maintaining relevant cognitive structures representing the situation at hand, and this eventually results in an enhanced SA and decision-making, and ultimately in actions.

With some exceptions, the results from this study are in line with the SMM theory and expand previous knowledge by empirically showing that shared mental models are the most decisive mechanism for team performance when the team approaches unclear, dangerous and difficult situations. This finding gives strong indications as to what type of training is necessary for teams that intend to cope in stressful environments.

### Big Five and Trainable?

We aimed at investigating whether Big Five teamwork and the coordinating mechanisms are trainable. In doing so, we relied on a brief training program that had previously been reported to have an effect on subjective learning as well as being relevant to operational scenarios (Johnsen et al., 2016). The scenario-based training gave the participants new experience and opportunities to identify knowledge gaps on which he/she could reflect (concrete experience) and to try out new ways of coping. Although the training program has shown to be effective both on subjective ratings of SA and target handling (Saus et al., 2021), no effect was found for the variables generated from the big five model using external SMEs as evaluators.

Even briefer training interventions than in this study have shown effect. For instance, Israel et al. (2014) reported a positive effect from a 5-h training program aimed at making law enforcement personnel work more effectively when meeting sexual minorities. However, this was on an individual level, and it is fair to suggest that the team context makes training more complicated, and therefore more time is needed. Saus et al. (2021) have shown the effects on SMEs' ratings of more non-specific measures of teamwork, such as internal and external communication, and the dynamic positioning of the team members relative to each other in target handling. However, the present study showed no effects when teamwork was measured as a specific theory-derived behavior. It is also possible that other intra-social mechanisms offset learned teamwork behaviors. For example, the composition of teams using police officers unfamiliar to each other and coming from different police units may have been subject to effects of key characteristics described in social identity theory (Tajfel and Turner, 1986). The passing of time between the training intervention and the test may also have played a part, as this was between 2 and 6 months. Apart from time, there could also be other explanations, such as content, design, or a lack of motivation for training.

We would argue that an 8-h training program is too short in which to learn and master complicated cognitive mechanisms such as mental models and to make them shared. Therefore, future research should devote more time and effort to the training interventions, and concentrate on cognition understood as SMM, and connect these to the Big Five teamwork behaviors.

### Limitations

Some caution should be noted regarding the high intercorrelation values obtained. Although the collinearity diagnostics stated that the variables were within an acceptable range, it could be that the subject matter experts treated the Big Five teamwork behaviors, coordinating mechanisms, and the performance indicators as similar concepts when they evaluated the police patrols. The evaluation was based entirely on SMEs' consensus ratings. This could also cause a problem since there is no measurement of variability between the raters, which results in a lack of reliability testing of the rating system. The procedure whereby two experienced police officers should agree on the score was intended to increase the possibility of differentiating between the concepts and to make the score more reliable and valid. Also, neither during the execution of the testing nor in the “hotwash” with the SMEs and the role players after the testing, did the variation of scores turn up as a problematic issue.

Multiple-item scales are favored to measure psychological constructs (Nunnaly, 1967), and this study relies on single-item measures. However, Wanous et al. (1997) and others support the use of single items. This is founded on empirical data showing high test-retest reliability (Littman et al., 2006), as well as high correlations with multiple-item scales (Wanous et al., 1997). The validity is also revealed by single-item measures effectively predicting outcomes (Nagy, 2002). Although there are limitations, potential advantages should be noted for the use of single items. These include cost-efficiency, greater face validity, and a possible increased willingness of respondents to take time to complete the questionnaire instigated by a less intrusive method compared to the use of multi-item scales.

Another limitation was the lack of observations during the driving or planning phase. However, the use of SMEs as raters made it possible to take into consideration part of the consequences of planning, such as (for example) their performance relative to their chosen equipment.

## Conclusions

To sum up, correlational and regression analyses of police patrols indicate that all “Big Five” teamwork behaviors and coordinating mechanisms are connected with external ratings of team performance indicators in stressful operational situations. Therefore, both the three coordinating mechanisms and the five team processes derived from the Big Five theory were related to increased performance. The study showed that only CLC and SMM predicted team performance in a regression analysis, with SMM predicting above and beyond the effect of CLC. Contrary to this, trust did not explain variance in team performance, which was interpreted as being caused by a generally high level of trust within the police force. The study provides new and strong evidence of SMM as the most important underlying factor for the Big Five theory. No effect of the training program occurred, since the trained group did not show more of the Big Five teamwork behavioral markers, nor better performance compared to the untrained group. This may be due to the 8-h training program being too short in order to learn and master complicated cognitive mechanisms such as SMM, or because other intrasocial mechanisms (e.g., social identity theory) offset any potential learned teamwork behavior during short, critical, and high-intensity scenarios.

## Data Availability Statement

The raw data supporting the conclusions of this article will be made available by the authors, without undue reservation.

## Ethics Statement

The studies involving human participants were reviewed and approved by NSD—Norwegian centre for research data. The patients/participants provided their written informed consent to participate in this study.

## Author Contributions

RE conceived of the presented idea, developed the theory, and planned the experiments. RE and BJ processed the experimental data, performed the analysis, drafted, wrote the article, and designed the figures. All authors carried out the experiment, discussed the results, and commented on the manuscript.

## Conflict of Interest

The authors declare that the research was conducted in the absence of any commercial or financial relationships that could be construed as a potential conflict of interest.

## Publisher's Note

All claims expressed in this article are solely those of the authors and do not necessarily represent those of their affiliated organizations, or those of the publisher, the editors and the reviewers. Any product that may be evaluated in this article, or claim that may be made by its manufacturer, is not guaranteed or endorsed by the publisher.
